# Practices of entomophagy and entomotherapy by members of the Nyishi and Galo tribes, two ethnic groups of the state of Arunachal Pradesh (North-East India)

**DOI:** 10.1186/1746-4269-7-5

**Published:** 2011-01-14

**Authors:** Jharna Chakravorty, Sampat Ghosh, Victor Benno Meyer-Rochow

**Affiliations:** 1 Biochemical Nutrition Laboratory, Dept. of Zoology, Rajiv Gandhi University, Arunachal Pradesh 791112, India; 2 School of Engineering and Science, Jacobs University, Research II (rm. 37) D-28759 Bremen, Germany

## Abstract

We prepared a consolidated list of edible and therapeutic insects used in Arunachal Pradesh (N.E. India) by two tribal societies (i.e., the Nyishi of East Kameng and the Galo of West Siang). The list is based on thorough, semi-structured field-interviews with 20 informants of each tribal group. At least 81 species of local insects, belonging to 26 families and five orders of insects, namely Coleoptera (24 species), Orthoptera (17 species), Hemiptera (16 species), Hymenoptera (15 species) and Odonata (9 species), are being used as food among members of these two indigenous societies. However, Nyishi use overall more species of insects as food than Galo people do and consume mostly Coleoptera and Hemiptera; amongst the Galo, on the other hand, Odonata and Orthoptera dominate. The selection of the food insects amongst the Nyishi and Galo is dictated by traditional tribal beliefs as well as the taste and availability of the insects. Depending on the species, only particular or all developmental stages are consumed. Some food insects may be included in the local diet throughout the year, others only when seasonally available. Commonly specimens are being prepared for consumption by roasting, frying or boiling. Twelve species of insects are deemed therapeutically valuable by the locals and are being used by the tribes investigated to treat a variety of disorders in humans and domestic animals. Members of the Galo use a greater number of insect species for remedial purposes than the Nyishi. With the degradation of natural resources, rapid population growth, and increasing influence of 'westernization', the traditional wisdom of entomophagy and entomotherapy is at risk of being lost. There is thus an urgent need to record the role insects play as components of local diets and folk remedies and to assess insect biodiversity in the light of these uses.

## Introduction

The term entomophagy refers to the use of insects as food. Insects represent a traditional food category in many cultures of the world. Insects, as the most species-rich taxon of all animals, exhibit an enormous biodiversity and represent a colossal biomass in Nature. According to Bodenheimer [1] they have played an important part in the history of human nutrition in Africa, Asia and Latin America. Detailed information regarding diversity, mode of consumption and economic value of edible insects in all tropical and subtropical regions of the world has been compiled by De Foliart [2], Nonaka [3] and Mitsuhashi [4]. Van Huis [5] has reported that there are approximately 250 highly nutritious, edible insect species in sub-Saharan Africa, Ramos-Elorduy [6] has registered around 535 edible species in Mexico, and Mitsuhashi [4] arrived at a figure of at least 1,900 species of edible insects worldwide.

Preference given to insect species utilized as food by humans, depends on the insect's palatability, availability, and nutritional value as well as on local traditions and customs. Besides being described by many insect enthusiasts as a tasty food commodity of high nutritive value, many insects are also considered to possess health-enhancing properties. In many parts of the world, different sections of the society have been using medico-entomological drugs to this day in their lives. A number of studies has in recent years drawn attention to the therapeutic value of certain species of insects, their products, and their developmental stages [7-15]. According to Pemberton [16] arthropods as parts of folk medicinal remedies continue to be important in China and Korea. In India the bee product honey is being used in several Ayurvedic formulations since time immemorial and Yamakawa [17] has shown that insects, generally, can be regarded as a source for the development of drugs with immunological, analgesic, antibacterial, diuretic, anaesthetic, and anti-rheumatic properties.

Traditional ethnobiological knowledge and the habit of accepting insects as food and as an integral part of local therapies is nowadays confined to the traditionally living, largely indigenous societies of regions that until now have experienced only a limited amount of 'westernization'. The therapeutic uses of insects are often a closely guarded secret and only passed on to certain individuals from one generation to another by word of mouth. Transfer of knowledge in this way is an age-old practice and a well accepted socio-cultural attribute among the ethnic societies of North-East India.

Most of the edible insects, some of which are crop pests, but at the same time possess high nutritional qualities, constitute an important part of the local daily diet and, stressed by Reim [18] and Meyer-Rochow [19], are not an emergency food accepted only during times of starvation. Some insect species, moreover, find use in various home remedies. Information on this aspect of local life anywhere in the world is very fragmentary and for North-East India has only quite recently become an issue of scientific inquiry [20-23]. The aim of the present study, therefore, is to expand the earlier research on edible and therapeutic species of insects to include some tribes of Arunachal Pradesh, not investigated earlier) in our survey of insect uses by humans in North East India.

Arunachal Pradesh, the largest state in North-East India, lies between 26° 28' and 29° 30' N latitude and 90° 30' and 97°30' E longitude and biogeographically is situated in the Eastern Himalayan province, a territory characterized by a complex system of mountains and valleys of variable elevation (50 to 7000 m). By virtue of its geographical position, climatic zones and altitudinal variations, the state's biodiversity is rich with large tracts of tropical, wet evergreen forests and subtropical, temperate and alpine vegetation. It is regarded a global biodiversity hot spot [24] and one amongst 200 identified, globally important eco-regions [25]. The state has a low population density of only 13/km^2 ^[26]. The state is not only biologically diverse, but is furthermore home to a rich diversity of traditional communities with 26 major tribes and 110 subtribes. These various communities with their local biological resources have a considerable understanding of Nature and thus possess deep ethno-biological knowledge. The tribes are totally dependent for their livelihood on the forests and their resources and collecting certain plants and animals for food and folk medicinal purposes has been an age-old practice for them. Among the tribal communities of Arunachal Pradesh, the Nyishi and Galo are some of the more prominent tribes. By initially focusing on these two tribes for fear that before long information on their uses of insects as food and medicine might no longer be available, we continue the earlier work on uses of insects as food and medicines amongst North-East Indian tribals summarized by Meyer-Rochow in 2004 [21]. Our future and ultimate aim is to present an inventory of the various uses of insects for all major tribes in North East India.

## Materials and methods

Extensive field surveys to record the various uses of insects amongst members of the Nyishi and Galo tribe were carried out in the two respective districts of East Kameng and West Siang in the north-east Indian state of Arunachal Pradesh. Ten villages, selected at random, were visited in each of the two tribal areas. The number of households per village was 12 - 20 (one village had 30). Frequently at least 2 houses were unoccupied, because the families had moved into the towns in search of work. At least two households inhabited by village elders and their families were visited. Recommendations by the headman or village elders to visit certain knowledgeable persons in another village were sometimes followed. The surveys were based on interviews during which a total of 20 persons aged between 45 and 70 years of age (12 male and 8 female) from each tribe were shown museum specimens or photographs of insects. The interviewed people were then asked simple questions in order to obtain information on the vernacular names of the edible or otherwise important insects, on seasonal availabilities, stages of insects consumed or used, mode of preparation, assumed therapeutic value, folklore related to insects and anything else deemed important in connection with the insect in question. As the knowledge of Hindi or English of the locals was often not great, our questions had to be simple and to the point.

Insects were collected from different habitats, e.g., ponds and streams, soils and farmland, shrubs and trees, grassland and dwellings. They were then preserved according to standard methods [27] and identified with the help of published keys [28-31]. Where this was not possible, the insects were sent to Kolkata to be identified by entomological experts of the Zoological Survey of India.

### Target Groups (Figure 1)

**Figure 1:**
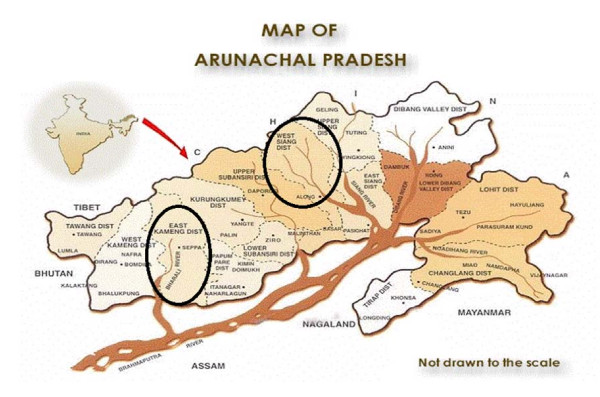
Map of Arunachal Pradesh (North-East India)

Members of the Galo tribe, inhabiting the West Siang district of Arunachal Pradesh located at 94°02'E - 95°15'E and 27°29' N-29°23'N at altitudes between 60 - 5000 m. The region's climate is markedly continental in character with average annual rainfall of 3000 mm and temperatures ranging from 5°C in the month of December to 38°C in the month of July. The total population of the district, which is inhabited by not only members of the Galo, but also Adi, Memba and Khamba tribes, is approximately 105,000. Galo people make up roughly one fourth of the population and are found in the southern part of this district. Traditionally Galos practiced shifting cultivation, but nowadays wet rice and terrace cultivations have become common.

Nyishi people inhabit the East Kameng district of Arunachal Pradesh located at 92° 36' E - 93° 24' E and 26° 56' N - 27° 59' N. The district is shared by people of the Sulung, Aka, Miji, and Bangni Nyishi. This study explores only the ethno-entomological knowledge of the Bangni Nyishi and Sulung, designated, however, together as Nyishi in the following text, because their inextricably linked life-styles. The temperature of the region ranges from 17°C in the month of December to 27°C in the month of July and the altitude range is 360 - 1900 m. Total population is ca. 57,000 and literacy rate is 41%; population density 14/km^2^. The major part of the area inhabited by Nyishis is covered by forests, cropped areas being very limited. Settled agriculture is yet to take on in a sizeable manner and only 0.03% of the area of the district is under regular cultivation.

## Results and Discussion

Details on the taxonomic position as well as the vernacular names are given for insect species that are consumed by members of the two ethnic groups. The information with regard to edible species and the assumed therapeutic uses of insects was considered only reliable, when it came from at least 40% of the respondents. Despite this precaution, we do not consider this study to be a quantitative one and, therefore, wish to stress that this is a qualitative study and the first of its kind for any Arunachal Pradesh tribe.

An inventory on what is known to date on the edible insects of the two ethnic tribes of the studied areas is presented in Tables 1, 2, 3, 4 and 5. The present study revealed that a total of at least 81 species of local insects (including both identified and non identified species), belonging to 26 families and five orders, finds acceptance as food by the locals. Out of the 81 species, 9 are representatives of the Odonata, 17 of the Orthoptera, 16 of the Hemiptera, 15 of the Hymenoptera and 24 of the Coleoptera. Silk worms, sold at local markets, are being consumed, but were not entered into our list, because they were not locally grown and brought into the region from outside the district. Although some species belonging to orders like Ephemeroptera and Mantodea were mentioned by some of the people questioned by us, these species are also not included here, because the number of respondents mentioning these insects was considered to be too low to have the species registered as being regularly eaten. However, we cannot, of course, rule out that certain species of insects are fancied by some specific subsection of the population, are subject to food taboo restrictions [32] or eaten under particular conditions, so that in the future as more detailed information surfaces our list of edible insects is likely to grow in length.

**Table 1:** Inventory of edible Odonata

Scientific name	Family	English name	Vernacular name	Seasonal availability	Mode of intake	Remark
*Enallagma *sp.	Coenagrionidae	Azure bluet	Esh tat tani (G) Soko yoyo (N)	Sept-Oct	Larval stages are preferred Raw with bamboo shoot. Wings are discarded when adults are consumed. when adults are	Found near swampy areas. Not consumed by Nyishi people.

*Ictinogomphus rapax*	Gomphidae	Clubtail dragon fly	Esh tat tani (G) Soko yoyo (N)	Sept-Oct	Larval stages are preferred. Raw with bamboo shoot. Wings are discarded when adults are consumed.	Found near swampy areas. Not consumed by Nyishi people.

*Stylurus *sp.(?)	Gomphidae	Clubtail dragon fly	Ani asum (Larval form)(G) Yash kori (N)	Jan-Mar	Nymphs boiled, raw and as roasted paste. Nothing is discarded	Amongst flowering aquatic plants and in rivers and along banks.

*Sympetrum *sp.	Libellulidae	Cardinal meadow hawk	Esh tat tani (G) Soko yoyo (N)	Sept-Oct	Raw with bamboo shoot. Larval stages are preferred. Wings are discarded when adults are being consumed.	Found near swampy areas. Not consumed by Nyishi people.

*Brachythemis contaminata*	Libellulidae	Ditch jewel	Esh tat tani (G) Soko yoyo (N)	Sept-Oct	Larval stages are most preferred. Raw with bamboo shoot. Wings are discarded when adults are consumed.	Found near swampy areas. Not consumed by Nyishi people.

*Urothemis *sp.(?)	Libellulidae	Dragon fly	Esh tat tani (G) Soko yoyo (N)	Sept-Oct	Larval stages are most preferred. Raw with bamboo shoot. Wings are discarded when adults are consumed.	Found near swampy areas. Not consumed by Nyishi people.

*Pachydiplax *sp. (?)	Libellulidae	Blue dasher	Esh tat tani (G) Soko yoyo (N)	Sept-Oct	Larval stages are most preferred. Raw with bamboo shoot. Wings are discarded when adults are consumed.	Found near swampy areas. Not consumed by Nyishi people.

*Diplacodes *sp.	Libellulidae	Chalky percher	Soko yoyo (Yare) (N)	Perennial	Larval stages are considered edible. Roasted	Not consumed by Galo people.

Unidentified			Esh tat tani (G) Soko yoyo (N)	Sept-Oct	Raw with bamboo shoot. Larval stages are edible. Wings are discarded when adults are consumed	Found near swampy areas. Not consumed by Nyishi people.

? to be confirmed here in Odonata. Mostly the larval stages are consumed. However, adult stages may also be consumed depending upon the catch.

**Table 2:** Inventory of edible Orthoptera

Scientific name	Family	English name	Vernacular name (G = Galo; N = Nyishi)	Seasonal availability	Mode of intake	Remark
*Chondacris rosea*	Acrididae	Short horned grasshopper	Mirbo (G) Takam kamrak (N)	Sept-Nov	Adult stage is consumed boiled, fried, or as paste (chutney). Some insects are smoked for further use. Wings, appendages and lower portion of abdomen are discarded.	Economically important, because of its taste and palatable size.

*Heiroglyphus *sp.	Acrididae	Short horned grasshopper	Mirbo (G) Eshi tech takam (N)	Sept-Dec	Adult stage is consumed. Fried, used with boiled vegetables or paste (chutney) to take with local alcohol/beverage. Antennae and appendages are discarded.	Highly esteemed by all age groups. Collected from paddy fields.

*Diabolocanthops innotabilis*	Acrididae	Clown grasshopper	Ili konkam (G) Timi kamchi (N)	Sept-Oct	Adult stage is consumed. Fried and boiled or smoked. Antennae and wings are discarded.	Moderately liked by all age groups.

*Schistocerca *sp.	Acrididae	locust	Komak joba(G) Takam soik (N)	Sept-Nov	Adult stage is consumed. Fried and boiled with some leafy vegetables. Wings, antennae, appendages and lower portion of abdomen (supposed to contain intestinal parasite) are discarded.	Esteemed by all age groups.

*Leptysma *sp. (American genus, but given as such by Zool Survey of India, Kolkata)	Acrididae	Short horned grasshopper	Takam hilak (G) Takam pario tokcho (N)	Aug-Oct	Adult stage is consumed. Boiled, roasted and paste is made. Anal cirri and antennae are discarded.	Esteemed by all age groups.

*Brachytrypes *sp. (African genus, but given as such by Zool Survey of India, Kolkata)	Gryllidae	Cricket	Yarup (G) Takam Yarup (N)	Sept-Nov	Adult stage is consumed. Fried, used in boiled vegetables or as paste (chutney) to take with local alcohol. Antennae and limb appendages are discarded.	Highly preferred by all age groups.

*Tarbinskiellus orientalis*	Gryllidae	Cricket	Komdruk (G)	May-Sept	Adult stage is consumed. Fried or roasted.	--

*Gryllotalpa *sp.	Gryllotalpidae	Mole cricket	Yarup (G) Takam gajir (N)	Sept-Dec	Adult stage is consumed. Boiled, roasted and as paste. Nothing is discarded.	This insect is also used as bait to catch fowl, bird, fishes, etc.

Unidentified	Phaneropteridae	Round headed katydid	Kombuk (G) Atu rungne (N)	Sept-Oct	Adult stage is consumed. Fried, paste (chutney) is made and boiled. Antennae are discarded.	A very sought after food insect; liked by everyone.

*Schizodactylus monstrosus*	Schizodactylidae	Sand cricket	Nyanyir (G) Ayu pokung (N)	May-July	Adult stage is consumed fried or roasted.	Not consumed by Nyishi people.

*Conocephalus *sp.	Tettigoniidae	Katydid	Kombuk (G) Atu rungne (N)	Sept-Oct	Adult stage is consumed fried or as paste (chutney) and boiled. Antennae are discarded.	A highly esteemed food insect for everyone.

*Arachnacaris *sp.	Tettigoniidae	Katydid	Kombuk (G) Atu rugnu hoie (N)	Sept-Oct	Adult stage is consumed fried and boiled or roasted. Appendages and antennae are discarded.	Preferred by children.

*Microcentrum *sp. (American genus, but given as such by Zool Survey of India, Kolkata)	Tettigoniidae	Katydid	Abo ngomdir (G) Takam yash (N)	Aug-Oct	Adult stage is consumed. Boiled with vegetables. Wings are discarded.	Consumed by all age groups, but no by Nyishi people.

*Chloracris brullei*	Tettigoniidae	Katydid	Komle (G) Paie kamge (N)	Sept-Oct	Adult stage is consumed boiled or as paste. Wings and antennae are discarded.	Consumed by all age groups.

Unidentified	Tettigoniidae	Katydid	Mir apo (G) Kamar dodar (N)	Sept-Oct	Larval form is consumed. Paste is made along with dried bamboo shoot.	Larvae are preferred to adults.

Unidentified	Tettigoniidae	Katydid	Yan pedak (G) Atu rungne (N)	Aug-Nov	Adult stage is consumed boiled and roasted. Wings and appendages are discarded.	Adults are preferred to larvae.

Unidentified			Kompe rene (G) Eshi tech takam (N)	Aug-Oct	Adult stage is consumed fried and boiled. Antennae to be discarded.	Consumed by all age groups.

**Table 3:** Inventory of edible Hemiptera

Scientific name	Family	English name	Vernacular name (G = Galo; N = Nyishi)	Seasonal availability	Mode of intake	Remark
*Lethocerus indicus*	Belostomidae	Giant water bug	Isi tari	Round the year	Boiled or fried as adult consumed	--

*Tibicen pruinosus *(American species, but given as such by Zool Survey of India, Kolkata)	Cicadidae	Annual cicada	Nyani (G) Laptung bargi (N)	Apr-June	Adult stage is consumed. Boiled or as paste. Wings are discarded.	Diurnal singer; stops menstrual cycle if taken in larger quantities.

*Cyclochila virens *(Australian species, but given as such by Zool Survey of India, Kolkata)	Cicadidae	Greengrocer cicada	Nyare tasi (G)	May-Aug	Adult stage is consumed. Roasted or as paste. Wings are discarded.	Diurnal singer. Not consumed by Nyishi.

*Euterphosia crowfooti*	Cicadidae	Cicada	Gopu goye (G) Yadung nengne (N)	May-July	Adult stage is consumed. Roasted or as paste. Wings are discarded.	Diurnal singer.

*Pycna repandar*	Cicadidae	Cicada	Gopu goye (G) Yato rugne (N)	May-July	Adult stage is consumed. Roasted or as paste. Wings are discarded	Diurnal singer.

*Aspongopus *sp.	Pentatomidae	Stink bug	Rishu (N) Rishu punyo (G)	Dec - Feb	Adult stage is consumed. Fried or boiled with vegetables.	--

*Alcaerrhynchus grandis *(American species, but given as such by Zool Survey of India, Kolkata)	Pentatomidae	Stink bug	Rishu (N) Rishu punyo (G)	Dec - Feb	Adult stage is consumed. Fried or boiled with vegetables.	Not consumed by Galo people.

*Tessaratoma quadrata*	Tessaratomidae	Stink bug	Tari (G) Agu chena rekok (N)	Feb-Mar	Adult stage is consumed. Raw or turned into chutney. Wings are discarded.	Large insect; considerable knowledge is required to avoid poisonous mimic. Not consumed by Nyshi as the bug bites and may cause fever.

*Halyomorpha picus*	Pentatomidae	Stink bug	Tari (G) Rishu (N)	Nov-Feb	Adult stage is consumed. Raw paste (chutney) is made. Head or in some cases abdomen is discarded.	

*Aspongopus nepalensis*	Pentatomidae	Stink bug	Tari Gondhi bug/gondhipuk (G, N)	Nov-Feb	Adult stage is consumed. Part of abdomen is discarded to avoid pungent taste; raw or as chutney.	Excessive consumption causes hallucination.

*Nezara viridula*	Pentatomidae	Stink bug	Rishu (N) Rishu punyo (G)	Dec - Feb	Adult stage is consumed. Fried or boiled with vegetables.	Not consumed by Galo people.

Unidentified	Pentatomidae	Stink bug	Rishu (N)	Dec - Feb	Adult stage is consumed. Fried or raw paste is made.	--

*Dalader acuticosta*	Coreidae	Plant bug	Rishu (N) Rishu punyo (G)	Dec - Feb	Adult stage is consumed. Fried or raw paste is made.	Causes burning sensation in body. Not consumed by Galo people.

*Mictis tenebrosa*	Coreidae	Plant bug	Rishu (N) Rishu punyo (G)	Dec - Feb	Adult stage is consumed. Fried or raw paste is made.	Not consumed by Galo people.

*Antilochus coqueberti*	Pyrrhocoridae	Red bug	Rishu (N) Rishu punyo (G)	Dec - Feb	Adult stage is consumed. Fried or boiled with vegetables.	Not consumed by Galo people.

Unidentified		Bug	Rishu (N) Rishu punyo (G)	Dec - Feb	Adult stage is consumed. Fried or boiled with vegetables.	Not consumed by Galo people.

**Table 4:** Inventory of edible Hymenoptera

Scientific name	Family	English name	Vernacular name (G = Galo; N = Nyishi)	Seasonal availability	Mode of intake	Remark
*Vespa *sp.	Vespidae	Wasps	Rego (G) Pacha yadam (N)	Nov-Jan	Adult forms are preferred. Fried, fresh one is chewed, wings are discarded. Though larvae pupae are also consumed	Fresh insects are chewed and chitinous discarded material is collected and used for metallurgical processing. Not consumed or used by Nyishi.

*Polistes *sp.	Vespidae	Potter wasp	Bere (G) Hupu hum yalang (red) (N)	Nov-Feb	Adult forms are preferred. Fried, fresh is chewed, wings are discarded, but larvae and pupae are also consumed.	Used in metallurgical processing.

*Polistes *sp.	Vespidae	Paper wasp	Oye nigona (G) Hupu hum yalang (N)	Nov-Feb	Larvae are collected along with bee hive and then smoked. Adult and larvae both are preferable, in case of adult wings are discarded.	--

Unidentified	Vespidae	wasp	Iddum (G) Tee (N)	Nov-Jan	Adult forms are preferred. Roasted, boiled, smoked or paste is made, wings are discarded.	--

Unidentified	Vespidae	wasp	Iddum ago (G) Hoie (baby of tee) (N)	Nov-Dec	Both adult and larval form are consumed though larval form is preferred a lot Fried or boiled, wings are discarded.	Larval stage is preferred.

Unidentified	Vespidae	wasp	Iddum tupte (G) Tatang (N)	Oct-Dec	Adult stage is consumed. Boiled with bamboo shoot, wings are to be discarded.	Adults are preferred

Unidentified	Ichneumonidae	Ichneumonid wasp	Bere (G) Gacha ganga hoie (young) (N)	Oct-Dec	Adult stage is consumed. Fried and paste is made, wings and terminal end of appendages are discarded.	Esteemed by all age groups.

*Eumenes *sp.	Vespidae (Eumenidae)	Potter wasp	Ite paglum (G) Gacha ganga hoie (mother) (N)	Nov-Dec	Larvae are eaten directly. Pupae stage is boiled or paste is made.	Larval form is highly preferred.

Unidentified	Vespidae	Wasp	Rele botu (G) Taga (N)	Dec-Feb	Egg, larvae, pupae, and adult stages are consumed. Egg/larvae are dried and boiled or turned into a paste; adults have wings discarded before consumption.	Egg/larvae most preferred.

*Vespa orientalis*	Vespidae	Wasp	Gapu (G) Gunya (N)	Nov-Feb	Larvae are collected along with nest and smoked; wings are to be discarded in case of adult insect consumption.	Larval stage is highly esteemed.

*Apis cerana*	Apidae	Honey bee	Tangik, (G) Tungu (N)	Nov-Jan	Adult and larval stages are consumed roasted and in form of a paste. Wings and antennae are discarded.	Preferred by all age groups.

*Apis *sp.	Apidae	Honey bee	Bere rusup (G)	Nov-Jan	Adult stage is consumed. After frying a paste is made and consumed with food; wings are discarded.	Not consumed by Nyishi.

*Xylocopa *sp.	Xylocopidae	Carpenter bee	Itum galum (G)	Nov-Mar	Adult and larval stages are consumed in boiled form; wings are to be removed in case of adult.	Not consumed by Nyishi.

*Oecophylla smaragdina*	Formicidae	Weaver ant	Tonge/Babuk (G) Babuk(N)	All year round	Adult and larval forms, both are consumed raw.	

**Table 5:** Inventory of edible Coleoptera

Scientific name	Family	English name	Vernacular name (G = Galo; N = Nyishi)	Seasonal availability	Mode of intake	Remark
*Sternocera *sp.	Buprestidae	Jewel beetle	Togum (G) Jorjo punyo (N)	June-July	Adult form is preferred. Boiled or smoked.	Not consumed by Galo people

*Oplatocera *sp.	Cerambycidae	Long horned beetle	Rigyo tapum (G) Sikse regret (N)	June-July	Adult form is preferred. Smoked, roasted or boiled. Wings and appendages are discarded.	Preferred by old people; may cause hair loss in adults. Not consumed by Galo.

*Aristobia *sp.	Cerambycidae	Long horned beetle	Anyo tapum (G) Sikse regre (N)	June-Aug	Adult form is preferred. Smoked, roasted or boiled. Wings are discarded	Not consumed by Galo.

*Batocera roylei*	Cerambycidae	Long horned beetle	Anyo tapum (G) Sikse regret (N)	June-Aug	Both larval and adult forms are taken. Smoked, roasted or boiled. Wings are discarded	Not consumed by Galo.

*Xylorhiza *sp.	Cerambycidae	Long horned beetle	Tani ane (G) Sikse regret (N)	June-Sept	Larval form is preferred. Boiled or fried.	

*Monochamus versteegi*	Cerambycidae	Long horned beetle	Sikse regret (N)	June-Sept	Adult form is preferred. Smoked, roasted or boiled. Wings are discarded.	Not consumed by Galo.

Unidentified	Cerambycidae	Long horned beetle	Anyo tapum (G) Sikse regre (N)	June-Aug	Adult form is preferred. Smoked, roasted or boiled. Wings are discarded.	Not consumed by Galo.

Unidentified	Cerambycidae	Long horned beetle	Anyo tapum (G) Sikse regre (N)	June-Aug	Adult form is preferred. Smoked, roasted or boiled. Wings are discarded.	Not consumed by Galo.

*Dorcus *sp.	Lucanidae	Stag beetle	Tonge lote (Male) (G) Tapu yagar nya (Male) (N)	Aug-Sept	Both larval and adult stages are preferred. Roasted, boiled or paste (chutney) preferred with alcohol. If consumed as adults, antennae and appendages removed.	Stem borer remains inside the bamboo shoot. Both adult and larvae are consumed.

*Prosopocoilus *sp.	Lucanidae	Stag beetle	Tonge ane (Female) (G) Tapu yagar nya (Male) (N)	July-Sept	Both larval and adult form are consumed. Larval stage is highly preferred because of high amount of fat content and its taste.. Adults are roasted and culminated with shoots of bamboo for intake, antennae and appendages are discarded.	_

*Odontolabis gazella*	Lucanidae	Stag beetle	Tonge (G) Tapu yagar nya (Male) (N)	July-Sept	Both larval and adult form are consumed. Larvae are fried slightly in oil and are added to boiled vegetables. It can be directly boiled with certain leafy vegetables. The larvae are rich in fat content. Appendages and antennae are discarded when adult is preferred.	Pest of bamboo, found inside bamboo shoot; can lead to death of whole bamboo plant

*Odontotaenius *sp.	Passalidae	Bess beetle	Esi nonge (G) Tapu yagar nym (N)	June-Aug	Both larval and adult form are consumed though larvae is preferred mostly than adult Roasted, smoked or boiled with vegetables (oiik). If boiled or fried wings are discarded.	In between ark and wood of tree.

*Polyphylla *sp. (or related genus)	Scarabaeidae	Scarab beetle	Tonge (G) Tapu yagar nym (Female) (N)	June-Aug	Both larval and adult forms are consumed though larvae is preferred than adult. Roasted. Antennae and appendages are discarded.	Pest of orange tree.

*Xylotrupes gideon*	Scarabaeidae	Rhinoceros beetle	Tonge (G) Tapu yagar nym (Male) (N)	May-July	Adult forms are preferred. Roasted, boiled	--

*Catharsius *sp.	Scarabaeidae	Cow dung beetle	Apo hunik (G) Ering dochu(N)	June-Aug	Adult forms are preferred. Wet paste is made and given to children during diarrhea. Body cover is discarded.	Not consumed by Nyishi people.

*Allomyrina dichotoma*	Scarabaeidae	Japanese rhinoceros beetle	Tapum (G) Rukching pungi (N)	June-Aug	Adult forms are preferred. Boiled, roasted and steamed for further use. Appendages are discarded.	Found on rotting bark amongst leaf litter on the ground.

*Lepidiota *sp.	Scarabaeidae	Christmas beetle	Apu nine (G) Tapu yagar nym (Female) (N)	Aug-Sept	Adult forms are preferred. Boiled or smoked.	Not consumed by Galo.

*Anomala *sp.	Scarabaeidae	Scarab beetle	Apu nine (G) Tapu yagar nym (Female) (N)	Aug-Sept	Adult forms are preferred. Roasted or boiled.	Not consumed by Galo.

*Propomacrus *sp.	Scarabaeidae	Scarab beetle	Sig re rigre (N)	June-Sept	Adult forms are preferred. Smoked, roasted or boiled. Wings are discarded.	Not consumed by Galo.

Unidentified	Scarabaeidae	May chafer	Hi tayabo (G) Jorjo punyo (N)	June-Aug	Adult forms are preferred. Smoked, boiled and roasted for culinary paste. If taken in boiled form wings are discarded.	Less preferred.

Unidentified	Scarabaeidae	Scarab beetle	Apu nine (G) Tapu yagar nym(Female) (N)	Aug-Sept	Adult forms are preferred. Smoked or boiled. Wings and appendages are discarded.	Not consumed by Galo.

Unidentified			Eh pako (N)	June-Sept	Adult forms are preferred. Smoked, roasted or boiled. Wings are discarded.	Found in bamboo plant. Not consumed by Galo.

Unidentified	Scarabaeidae	Chafer beetle	Apo hunik (G) Jorjo punyo (N)	June-Sept	Adult forms are consumed. Smoked, boiled and roasted for culinary paste. If taken in boiled form wings are discarded,	Found in large amounts, but less preferred.

*Trictenotoma *sp.	Trictenotomidae	Borer beetle	Sig re rigre (N)	June-Sept	Adult forms are preferred. Smoked, roasted or boiled. Wings are discarded.	Not consumed by Galo.

### Seasonal availability

Although edible insects generally occur throughout the year, their densities and diversities are determined by their food plants as well as by seasonal conditions. Observations on the seasonal availability (*cf*. Tables 2, 3, 4, 5) of the edible insects indicated that the maximum number of edible Coleopterans occurred during June to September (pre monsoon and monsoon) and then got reduced during winter and early spring. Seasonal trends were also observed in some Odonata and Orthopterans, which were most abundant in September and October (late summer). Insects belonging to the Hemiptera and Hymenoptera were found to be restricted to the period lasting from November to February (winter). Some edible insects like certain bugs and ants were found to be available (and used) throughout the year.

### Stages and modes of insect consumptions

Members of both tribes ate immature as well as adult stages of insects. However, in some cases, as with virtually all of the Odonata whose aquatic larvae were greatly preferred to the flying adults, only immature insects were consumed, but in others, as with the Orthoptera and Hemiptera the adult stages were more highly appreciated. Katydid species were an exception and preferred as wingless, immature specimens. Hymenopterans were eaten at all development stages: eggs, larvae, pupae and adults and even their products like honey, propolis, and wax were used. Most of the edible beetles were consumed as adults, although some like *Xylorhiza *sp. were clearly preferred in their larval stages. The beetles *Prosopocoilus *sp. and *Odontolabis gazilla *were consumed equally readily as larvae and adults. Preference for larval or adult stages almost certainly depended on a variety of factors: palatability of the insects (which may change between developmental stages), availability and the convenience with which the sought-after insects can be obtained, and furthermore taboos or religious beliefs may be involved. With regard to Odonata their aquatic larvae are clearly easier to collect than their adults and for Coleoptera with their wood-boring grubs the same would hold true.

Methods to prepare the edible insects for human consumption include roasting, boiling, or frying. Pentatomid bugs and honeybees, however, are being consumed both raw and roasted. Members of the two ethnic tribes interviewed by us explained that they possess various ways to improve the taste of an insect dish. Short-horned grasshoppers (Acrididae), for instance, are fried in oil after having their wings removed and are then simply eaten with salt. The insects, however, may also be stuffed in a bamboo pipe, smoked dry for 3-4 days, mixed with chili and salt and then added to rice meals. Long-horned grasshoppers (Tettigonidae), collected in smaller numbers than their short-horned cousins because of the solitary habits of the former, are roasted or fried in oil after having their wings removed. They are usually fed to children or aged persons.

Crickets and mole crickets (Gryllidae and Gryllotalpidae) are collected mostly during summer nights between the months of May and July. Yet the most highly valued orthopteran food insects amongst the Galo are Asian dune crickets of the species *Schizodactylus monstrosus *(Schizodactylidae). Freshly collected specimens are put inside a bamboo pipe and smoked dry for nearly one week. Completely dried material is then crushed into a powder and mixed with chili peppers, salt and bamboo shoots to form a special type of chutney. This chutney is taken with rice or with a local drink known as Apung and is regarded as most delicious by all members of the tribe irrespective of age and sex. Insect chutneys can also be based on other species, raw or dried, which are turned into a paste with chili and salt. Pentatomid bugs like *Aspongopus nepalensis *and other species, collected from river banks, are also highly appreciated in the form of a chutney by members of both tribes.

### Comparisons between the two tribes with respect to edible insects

**Figure 2:**
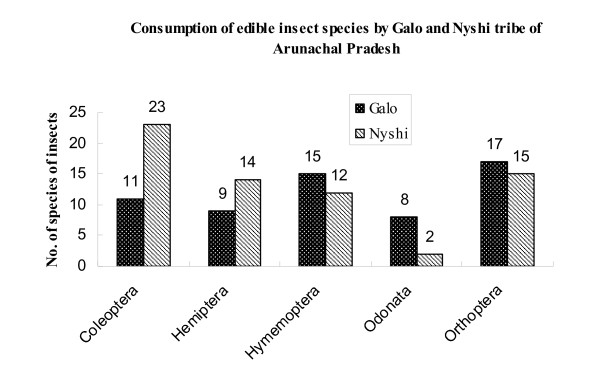
Numbers of insect species in 5 major orders consumed by members of the Nyishi and Galo tribes

Similarities and differences (Figure 2) of the entomophagy habits between the two tribal groups exist. In both tribes species belonging to five orders of insects are being consumed and modes of intake as well as stages of insects taken are quite similar between the two tribes. However, the total number of species consumed by the Nyishi Bangni of the East Kameng district is higher than that of the Galo of the West Siang district. In the West Siang district mostly Orthoptera followed by Hymenoptera and Odonata serve as food, but in the East Kameng district Coleoptera followed by Hemiptera are more frequently taken. The consumption of insects belonging to the remaining orders is rather similar between Galo and Nyishi: for the Orthoptera the figure was 17 : 15 species and for the Hymenoptera it was 15: 12. With regard to the consumption of Odonata, however, the difference was 8:2 between Galo and Nyishi, while the respective values for Hemiptera and Coleoptera were 9:14 and 11:23. The reason for these differences could be related to the Galo practice of wet rice and terrace cultivation, in other words to the different agricultural practices, which in case of the Galo provide environments especially conducive to the Orthoptera and Odonata. The territories, which the Nyishi inhabit are mostly covered by forests with cropped areas for agriculture limited to only 0.03%. Thus the forest environment combined with indigenous food acquisition practices are the major reasons for the greater consumption of Coleoptera rather than Orthoptera and Odonata by the Nyishi people of East Kameng.

During the field visits some Galo respondents explained that according to their belief system the use of Odonata by humans mirrors that of "the fishes that take them"; consequently these insects had to be good. The acceptability of insects as food by any indigenous society depends to a large extent on the traditions and beliefs of the society [32]. Species of the beetle genus *Batocera *are amongst the most widely accepted Coleoptera as food, being reported from Indonesia, Philippines, Sri Lanka and Papua New Guinea [33]. In the East Kameng district, Nyishi people consume *Batocera *spp. but in West Siang the Galo are not accepting these beetles. Some scarabaeid species, belonging to the genera *Lepidiota, Anomala*, and *Propomacrus *are consumed by Nyishi, but not Galo people. *Catharsius *sp. is one of the favourite insect food items of the Galo, but not the Nyshi people. Some of the pentatomid and pyrrhocorid bugs are rejected from the list of edible insects by the Galo, as the Galo believe these bugs are hallucinogenic, neurotoxic, allergenic and even fatal if ingested in large quantities. To avoid undesired reactions caused by the consumption of certain species of insects, sometimes highly specific preparation methods exist and frequently appendages that can cause some allergic reactions and, in the case of some bugs, parts of the abdomen that may contain hallucinogens or neurotoxins are removed by the Galo people. Obviously utilitarian principles are involved [34] and the fact that a food item, acceptable to one tribe, is rejected by the neighbouring tribe would remove, of course, pressure from the resource and makes good ecological sense [35].

### Comparisons between the two tribes with respect to insects assumed to possess therapeutic properties

The use of natural resources for therapeutic purposes is as old as humankind and continues around the world to this day. Ethnobiological knowledge has been passed on from generation to generation and one ought to expect that age-old practices valued to this day must be based on experience and fulfil a need. One part of our study, therefore, focuses on the traditional knowledge of insects with therapeutic properties. Although insects (species as well as individuals) are extremely numerous in Arunachal Pradesh, members of the various ethnic groups never collect and consume insects in a haphazard, random and unselective fashion, but follow unwritten rules and traditions. The traditions not only govern which species can be collected and taken as food, but extend to the insects' therapeutic uses. Species considered medicinally important by the Galo and Nyishi are listed in Table 6. The local people use the insects in home remedies not only for themselves but for their domesticated or semi-domesticated live stock as well.

**Table 6:** Inventory of Therapeutic insects

Scientific Name	English name	Vernacular Name (Galo = G; Nyishi = N)	Part used	Indication	Prescription	Remark
*Apis cerana, A. florae, A. mellifera *(Hymenoptera: Apidae)	Honey bee	Taer, Tang, Unya, Aati (G) Ngunya, Taer, Tangu (N)	Honey, comb Comb/wax	Honey: coughs, fevers, stomach pains, stomach cleanser. Skin irritations/disease	One spoon 2-3 times per day till completely cured Comb/wax Externally rubbed on skin irritation	Excessive use of honey causes adverse effect.
*Polistes *sp. *Vespa orientalis *(Hymenoptera: Vespidae)	Potter wasp	Bere, Taga/Gaying (G)	Whole insect, wings are removed	Coughs & colds, stomach disorders	Insects are directly allowed to bite or sting person suffering from cough & cold	Used in metallurgical process too (some respondents only).

*Bothroponera rufipes *(Hymenoptera: Formicidae)	Black ant	Yapek gane (G) Torup (N)	Whole body	**1**. Scabies, toothache; high blood pressure, boils, wounds malaria, dysentery, chest pain in humans **2**. Foot and mouth disease of mithun cattle (N) **3**. Maggots/worm infections of cattle	**1**. Ants are crushed into paste and applied on effected parts for scabies, wounds and boils. Ground-up ants are mixed with water and gurgled for some time for toothache. Intake of 1-2 ant per day reduces blood pressure. Intake of crushed ant along with other edibles during morning hour good for malaria. **2**. 1-2 ants crushed into powder and mixed into any kind of fodder and fed one to two times a day depending upon the persistence of the disease. **3**. Dried ants are mixed with warm water to wash the infected portion after removing the maggots from the wounds.	If fed in larger quantity, cattle becomes weak and inactive.

*Tetraponeraa rufonigra *(Hymeoptera: Formicidae)	Iron ant	Rukdam (G), Ruder (N)	Whole body	Foot and mouth disease of mithun cattle (N)	Fed 1-3 times per day according to persistence of disease. 1-2 ants crushed into powder and mix into any kind of fodder.	If fed in larger quantity cattle becomes weak and inactive. The effect of this species is much less than that of black ant.

*Oecophylla smaragdina *(Hymeoptera: Formicidae)	Red tree ant or weaver ant	Babuk (G, N)	Whole body	Stomachache and dysentry	One full colony is fried without oil; Smoked dried, mixed with salt and taken as small amount once a day till recovered.	_

*Oecophylla smaragdina *(Hymeoptera: Formicidae)	Red tree ant or weaver ant	Babuk aan (N) Tonge(G)	Whole body (N) Larvae (G)	Stomach pain, Fever	1 or 2 queen ants boiled and swallowed. Larvae are taken raw or boiled to lessen fever.	_

*Ephemera danica*	Mayfly		Nymph	Stomach disturbance	Roasted or boiled nymphs are consumed as food	

*Cantharid *sp. (Coleoptera: Cantharidae)	Beetle	Aputita (G)	Whole body	Skin allergy	--	_

*Lepidiota *sp. (Coleoptera: Scarabaediae)	Beetle	Aputita, Apu nine (G)	Whole body	Skin allergy	--	_

*Catharsius *sp. (Coleoptera: Scarabaeidae)	Beetle	Apu hanik (G)	Body cover is removed	Diarrhoea	Wet paste is made and given during acute diarrhoea	_

*Oplatocera *sp. (Coleoptera: Cerambycidae)	Longhorn beetle	Sikse regre (N)	_	_	_	It may cause hair loss in adults

*Chondracris rosea *(Orthoptera: Acrididae)	Grasshopper	Mirbo (G) Takam kamrak (N)	_	_	_	It shows allergic reaction in some people

*Diabolocantops innotabilis *(Orthoptera : Acrididae)	Grasshopper	Ili konkam (G)	_	_	_	Can cause allergy and initiate hair loss in some people

*Schistocera *sp. (Orthoptera: Acrididae)	Grasshopper	Komak joba (G)	_	_	_	Femur is most allergic part

*Brachytrypes *sp. (probably *Tarbinskiellus *sp. (Orthoptera: Gryllidae)	Cricket	Yarup (G)	_	_	_	Used as bait to catch fowl, bird, fishes etc.

Several species of pentatomid bugs (Hemiptera: Pentatomidae)	Pentatomid stink bugs	Rishu punyo (G)	_	_	_	Hallucinogenic, may cause allergies and some spp. considered neurotoxic

	Cicada		_	_	_	Not to be touched or killed by pregnant women. Thought to affect developing baby (baby starts crying in cicadal voice), but ok if both husband and wife touch or kill the insect together at the same time

*Tibicen pruinosus *(Hemiptera: Cicadidae) (American species, but given as such by Zool Survey of India, Kolkata)	Cicada	Laptung bargi (N)	_	_	_	Thought to stop menstrual cycle when consumed

(Hemiptera: Pentatomidae)	Pentatomid bug	Rishu (N)	_	_	_	Burning sensation occurs in body

During the field survey it was observed that the inhabitants of the most remote villages do not have much of a concept of diseases like diabetes, hepatitis, cancer to name but a few. They are mostly aware of coughs, colds, fever, stomach troubles, skin disorders, pains in the body and other obvious signs of illness. Therefore, our list is only based on the information given by the locals on diseases they recognize. We also observed that the locals prefer their own home remedies to medicines they do not know (and subsequently do not trust). It became obvious, however, that both tribes visited by us use very similar types of insect-based remedies (Table 6). Moreover, they both agree on which part or parts of an insect can be considered poisonous and this information is also presented in Table 6. As with the earlier inquiry into edible species, we considered the information given to us only sufficiently reliable and thus recordable when the same information came from at least 40% of the respondents. The one exception we allowed concerned the therapeutic use of mayflies, which we noted down even if only 35% of the respondents had mentioned it.

Our study identified twelve species of insects that were therapeutically used. Out of the twelve, eight species serve as the raw material for the treatment of two or more diseases. Species of the order Hymenoptera are the therapeutically most widely used insects, but the Coleoptera also feature with three medicinal species. Most of the therapeutic insects are taken raw or boiled and they are being used primarily to remedy stomach disorders, coughs and colds, skin allergies, boils, malaria, blood pressure anomalies, scabies (in case of humans) and foot and mouth disease of bovids like mithun and cattle. Galo as well as Nyishi make use of whole insects and not individual body parts, but the Galo use a greater variety of species than the Nyishi. The fact that Nyishi know more edible insect species than Galo people, but Galos value insects more from a therapeutic angle, confirms Meyer-Rochow's prediction of the greater persistency of therapeutic rather than dietary uses of insects [36].

The use of honey and bee's wax is common among members of both tribes to treat coughs and colds and apparently has a long history. Honey is considered to soothe the inflamed membranes of the mucus-secreting tissue of the upper respiratory tract and to relieve irritating symptoms that lead to difficulties in swallowing. Honey and wax are components of several Ayurvedic formulations, but whether their use entered Nyishi and Galo traditions from Ayurvedic teachings or was independently discovered by members of these tribes is unknown.

Wasps are also being used in the treatments of coughs, colds, and stomach disorders. Freshly killed wasps are meant to be chewed, but not swallowed, and said to provide strength to a patient. Wasps have also been reported as parts of the folk medicine of various Latin and South American cultures [37,38], as well sub-Saharan Africa, where they are often associated with strength on account of their sting [39]. Another medicinally-useful insect to the Nyishi and Galo is the blister beetle, which is said to help against skin allergies. As with the wasps, blister beetles, too, have in the past been used therapeutically in many parts of the world [16,38,40] and especially in Europe used to be a regular item on the shelves of pharmacies [41].

The use of ants amongst the Nyishi and Galo is also significant. These formic acid containing insects are being used in connection with scabies, malaria, tooth aches, stomach disorders, blood pressure anomalies, etc. in humans and foot and mouth disease as well as worm infections in cattle. Soil dwelling ants have been shown to produce compounds that kill both fungi and bacteria in their underground nests [42] and a paste made from termites (although not ants, but seen as related to ants by the local people) applied to an injured sheep or goat to speed up the healing of their wounds has been described from India [43]. Australian Aborigines, too, were apparently aware of the anti-inflammatory effects of crushed insects (in their cases cockroaches) rubbed into a wound [44] and western-trained surgeons even to this day are using maggots to clean up flesh wounds in both humans [45-47] and animals [48].

Sharma and Khan [49] observed that drugs of insect origin used by the tribal population of the Garo Hills of Meghalaya (N.E. India) are more common than those of mammalian origin. Although the reverse appears to hold true for the Galo and Nyishi tribes of Arunachal Pradesh, even here, as we have seen, insects play important roles in the treatment of disorders. As has been documented insects can be a source of drugs used in modern medicine, since compounds of insect origin can have immunological, analgesic, antibacterial, diuretic, anaesthetic and anti-rheumatic properties [17,50]. In fact, anti-microbial peptides were first discovered in insect larvae by Dr. Hans Boman of the Karolinska Institutet [51] and compounds, derived from chitin, have been used as anti-coagulants, become involved in the repair of various tissues on account of their effects on the auto-immune system, and even found an application in the fabrication of contact lenses [52].

The numerous chemicals that arthropods possess in their armament to defend themselves may either be products manufactured by them alone or represent derivatives of substances obtained from plants or prey [53-55]. It is interesting to note that arthropods and plants frequently utilize the same chemicals when repelling an attack against them [56]. It is likely that identical or at least similar compounds are also used by them to fight fungal, bacterial and viral infections. Since plants or their chemicals constitute one of our largest sources of drug material [57], it is reasonable to expect pharmacological activities from those arthropods that feed on drug-producing plants and allow their defensive substances to become concentrated in the arthropod's body. As the number of insect species used effectively by the Nyishi and Galo to treat certain afflictions indicates, traditionally-living tribals like them have apparently known this for generations.

## Conclusion

Unfortunately the availability of all types of modern food stuffs and the degradation of resources makes ethnic people worldwide (and the Galo and Nyishi are no exception) inclined to abandon their traditions and discard their rich indigenous knowledge. This is particularly lamentable in view of the fact that from a nutritional aspect, the traditional food is often not only healthier, it is also the product of generations of harmonious co-existence between tribe and environmental resource. The flipside of the coin is that due to unprecedented population increases, the resources of the forest, including food insects, can become over-exploited and this has apparently already resulted in the diminishment of biotic resources (including edible insects and species deemed therapeutically useful by the local people) in some parts of North-East India (Changkija 2010, personal communication). Although hard data are not (yet) available, many of our informants indicated that it has become increasingly more difficult to collect useful insect species. Therefore, we see an urgent need to assess insect biodiversity and the role of ethno-entomology together and not separated from each other. On the one hand, we feel that it is important to make sure that practices of entomophagy and entomotherapy do not disappear; on the other hand, we need to protect the biodiversity of the region and to conserve the valuable insect resources found in this region for posterity. Squaring these two demands (conservation of indigenous practices and prevention of over-exploitation of insects considered useful) must be a priority task for the future development of the region.

## Competing interests

The authors declare that they have no competing interests.

## Authors' contributions

JC carried out the field work and supervised SG's research. SG participated in the field work and identification of the insects. VBM-R began the ethno-entomological studies in North-East India and participated in the design, coordination, and drafted the final manuscript. All authors read and approved the final manuscript.
